# Hematopoietic Stem Cells Contribute to Lymphatic Endothelium

**DOI:** 10.1371/journal.pone.0003812

**Published:** 2008-11-26

**Authors:** Shuguang Jiang, Alexis S. Bailey, Devorah C. Goldman, John R. Swain, Melissa H. Wong, Philip R. Streeter, William H. Fleming

**Affiliations:** 1 Division of Hematology and Medical Oncology, Department of Medicine, Oregon Health & Science University, Portland, Oregon, United States of America; 2 Department of Dermatology, Oregon Health & Science University, Portland, Oregon, United States of America; 3 Oregon Stem Cell Center and Oregon Cancer Institute, Oregon Health & Science University, Portland, Oregon, United States of America; Texas Tech University Health Sciences Center, United States of America

## Abstract

**Background:**

Although the lymphatic system arises as an extension of venous vessels in the embryo, little is known about the role of circulating progenitors in the maintenance or development of lymphatic endothelium. Here, we investigated whether hematopoietic stem cells (HSCs) have the potential to give rise to lymphatic endothelial cells (LEC).

**Methodology/Principal Findings:**

Following the transfer of marked HSCs into irradiated recipients, donor-derived LEC that co-express the lymphatic endothelial markers Lyve-1 and VEGFR-3 were identified in several tissues. HSC-derived LEC persisted for more than 12 months and contributed to ∼3–4% of lymphatic vessels. Donor-derived LECs were not detected in mice transplanted with common myeloid progenitors and granulocyte/macrophage progenitors, suggesting that myeloid lineage commitment is not a requisite step in HSC contribution to lymphatic endothelium. Analysis of parabiotic mice revealed direct evidence for the existence of functional, circulating lymphatic progenitors in the absence of acute injury. Furthermore, the transplantation of HSCs into *Apc^Min/+^* mice resulted in the incorporation of donor-derived LEC into the lymphatic vessels of spontaneously arising intestinal tumors.

**Conclusions/Significance:**

Our results indicate that HSCs can contribute to normal and tumor associated lymphatic endothelium. These findings suggest that the modification of HSCs may be a novel approach for targeting tumor metastasis and attenuating diseases of the lymphatic system.

## Introduction

Functional lymphatics are critical for extracellular fluid homeostasis, fat absorption from the gut and immune function [1], [2], [3]. Lymphatic vessels also provide a route for leukocytes in tissues to re-enter venous circulation, and thus play an active role in acute and chronic inflammation. Importantly, tumor induced lymphangiogenesis has recently been shown to actively potentiate the metastatic spread of some cancers [2], [4], [5], [6]. Despite these important roles in normal and pathologic processes, only recently have we begun to gain an understanding of how the lymphatic system is maintained.

In the embryo, lymphatic endothelium arises from existing venous endothelial cells [7]. Although they share a common origin, lymphatic and venous endothelia are quite distinct at the morphological, functional and molecular levels. For example, in contrast to venous endothelium, lymphatic endothelium lacks a continuous basement membrane, is not surrounded by pericytes, and is largely devoid of vascular smooth muscle cell coverage [2]. Moreover, lymphatic endothelium highly expresses a number of proteins that are absent or expressed at relatively low levels in blood vascular endothelium. These lymphatic markers include the CD44 homolog, lymphatic endothelial hyaluronan receptor -1 (Lyve-1), vascular endothelial growth factor receptor-3 (VEGFR-3), Podoplanin and the homeobox transcription factor Prox1 [3].

The mechanisms by which new lymphatic vessel growth occurs in adults (i.e., lymphangiogenesis) and by which existing lymphatic vessels are repaired or remodeled after injury are currently not known. Previously, we [8], [9] and others [10], [11] demonstrated that adult bone marrow-derived, hematopoietic stem cells (HSCs) give rise to functional vascular endothelial cells in the mouse at the clonal level through differentiation in the absence of cell fusion. Furthermore, we [12] and others [13] have shown that in humans, hematopoietic derived cells contribute to both normal and tumor vascular endothelium. Taken together, these results indicate that adult bone marrow-derived hematopoietic stem cells may serve as a source of vascular endothelial progenitor cells. These findings raise the question of whether HSCs contribute to the maintenance and function of normal lymphatic endothelium (LEC).

Here we show that adult hematopoietic stem cells can give rise to LECs that integrate into lymphatic vessels in normal tissues and in newly formed tumors. By contrast, myeloid progenitors do not detectably contribute to LECs. We also demonstrate that the hematopoietic contribution to lymphatic endothelium can be mediated by circulating cells in the absence of acute radiation injury. These findings suggest a role for hematopoietic cells in the maintenance of lymphatic homeostasis.

## Results

### Evaluation of lymphatic vessel-specific markers

We focused most of our studies on mouse liver, specifically in the portal triad area (which contains the portal vein, hepatic artery, bile ducts, and small lymphatic vessels), because of the high frequency and distinctive morphology of the lymphatic vessels in this tissue. In order to distinguish lymphatic from blood vascular endothelial cells, we evaluated expression of the lymphatic markers Lyve-1 [14] and VEGFR-3 [15], in combination with the pan-endothelial cell marker CD31/PECAM-1, and the blood vessel endothelium-specific marker von Willebrand factor (vWF). As Prox1 is expressed by hepatocytes throughout the adult mouse liver [16], it was not utilized as a marker in our studies.

**Figure 1:**
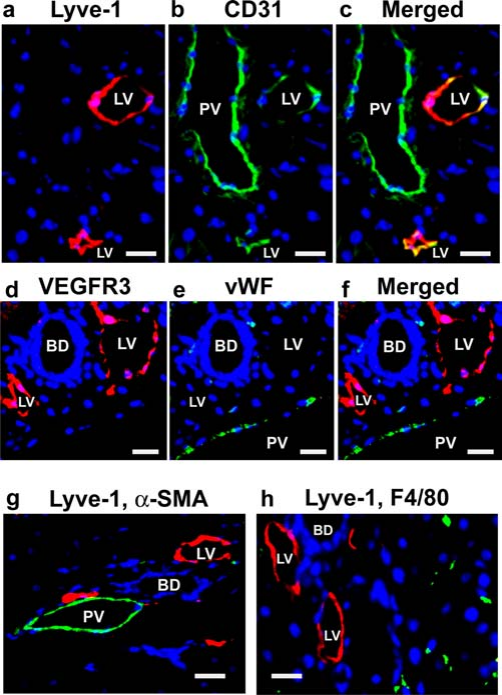
Lymphatic endothelial cells are readily distinguished from vascular endothelial cells. The portal triad region of mouse liver was analyzed for the expression of lymphatic (Lyve-1, VEGFR-3) and vascular (CD31, vWF) endothelial cell markers, a macrophage marker (F4/80) and a pericyte/smooth muscle marker (α-SMA). (a) Lymphatic endothelial cells are Lyve-1^+^ (red). (b) Both portal veins and lymphatic vessels express CD31 (green). (c) Merged image of a and b. Double positive CD31^+^, Lyve-1^+^ cells are yellow. d) VEGFR3^+^ lymphatic endothelial cells (red), do not express (e) the blood vessel endothelial marker vWF (green). (f) Merged image of d and e. (g) Lyve-1^+^ lymphatic vessels (red) do not express the pericyte marker α-SMA (green) or (h) the macrophage marker F4/80 (green). (DAPI stained nuclei are blue. PV = portal vein, LV = lymphatic vessel, BD = bile duct; scale bars: 20 µm).

In the portal triad, we readily identified vessels that were strongly immunoreactive for Lyve-1 (Fig. 1a) and co-expressed CD31 (Fig. 1b,c). By contrast, the portal vein (Fig. 1a,c) and other large blood vessels completely lacked Lyve-1 expression. Consistent with these findings, lymphatic vessels expressed VEGFR-3, but not vWF, whereas portal veins expressed vWF, but not VEGFR-3 (Fig. 1 d–f). As endothelial cells are often closely apposed to other cell types, it is critical to distinguish them from neighboring cells. Lymphatic vessels in the portal triad are devoid of smooth muscle actin (α-SMA) expressing pericytes and vascular smooth muscle, unlike blood vessels which are closely associated with these cell types (Fig. 1g). Macrophages are also known to closely associate with endothelial cells and express a number of endothelial cell markers. Notably, the Lyve-1 antigen is expressed by stabilin-1^+^, F4/80^+^, CD11b^+^ macrophages in tumors and in wound healing models [17]. Therefore staining of Lyve-1 and F4/80^+^ was employed to determine whether a similar Lyve-1^+^ macrophage population exists in the portal triad region of healthy mice during steady state conditions. We readily detected F4/80^+^ cells, however none co-expressed Lyve-1 (Fig. 1h). Taken together, our results confirm that lymphatic endothelial cells can be clearly distinguished from blood vessel endothelium, macrophages and pericytes by their morphology, location, high levels of Lyve-1 and VEGFR-3 expression, and absence of macrophage (F4/80), pericyte (α-Sma) and endothelial (vWF) cell –specific marker expression.

### Adult hematopoietic stem cells contribute to lymphatic endothelium in multiple tissues

**Figure 2:**
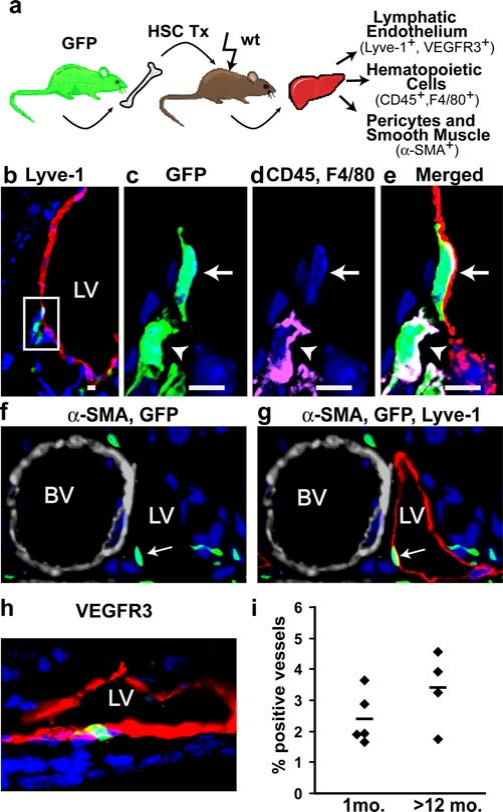
HSC-derived lymphatic endothelial cells stably incorporate into lymphatic vessels. a) Six months following transplantation of GFP^+^, c-kit^+^/Sca-1^+^/lin^−^ hematopoietic stem cells, recipient liver was examined for donor-derived lymphatic endothelium, hematopoietic cells, vascular smooth muscle and pericytes. (b) Low power view of donor-derived, GFP+ (green) cells (box) associated with a Lyve-1+ (red) lymphatic vessel (LV). (c–e) High magnification, confocal images of the boxed area shown in b. The arrow indicates a donor-derived lymphatic endothelial cell that is (c) GFP+ (green), (d) CD45^−^ and F4/80^−^ (absence of magenta), and is (e) Lyve-1+ (red). Nuclei are stained with DAPI (blue). The arrowhead indicates a donor-derived (green), CD45^+^ and/or F4/80^+^ (magenta) hematopoietic cell next to the lymphatic vessel. Cells in which GFP overlaps with DAPI and CD45 and/or F4/80 appear white (green+ blue+ magenta = white). Cells in which GFP overlaps with Lyve-1 (red+ green = yellow) and DAPI appear white (red+ green+ blue = white). (f & g) α-SMA expression (grey) distinguishes blood vessels (BV) from lymphatic vessels (LV). (f) Donor-derived cells (green) near a blood vessel surrounded by α-SMA^+^ pericytes (grey). The arrow points to a (g) Lyve-1+ (red) donor-derived cell within a lymphatic vessel. (h) A merged image showing a donor-derived GFP+ (green), VEGFR3+ (red) lymphatic endothelial cell in a lymphatic vessel. (i) Frequency of liver lymphatic vessels containing donor-derived endothelial cells 1 month to >12 months after HSC transplantation. Each symbol represents an individual HSC recipient. The horizontal line indicates the average of each group. (Panels b–e, scale bars are 5 µm.)

In order to evaluate the potential of adult hematopoietic stem cells to differentiate into lymphatic endothelium, between 200 to 500 sorted GFP^+^ c-kit^+^, Sca-1^+^, lineage^-^ (KSL) cells, hereafter referred to as HSCs, were transplanted into lethally irradiated (1200 cGy) recipient mice (Fig. 2a) as previously described [8]. Donor-derived cells in the peripheral blood and bone marrow of the chimeric mice were detected by flow cytometry, with GFP^+^ cells comprising between 80–90% of nucleated hematopoietic cells (data not shown). From one to 20 months following HSC transplantation, recipient tissues were harvested and evaluated for the presence of donor-derived lymphatic endothelium defined as GFP^+^ and Lyve-1^+^ and/or VEGFR-3^+^, CD45^−^ and F4/80^−^.

As shown in Figure 2b–e, CD45^−^ and F4/80^−^ GFP^+^ donor cells were readily detected in the lymphatic endothelium. Co-localized expression of endothelial markers in individual donor-derived GFP^+^ LECs was confirmed by confocal microscopy (Fig. 2c–e). As it is recognized that bone marrow derived cells also give rise to vascular pericytes and perivascular supportive cells [18], we assessed α-SMA expression in donor-derived cells incorporated into lymphatic vessels. No α-SMA co-expression was detected in these GFP^+^, Lyve-1^+^ cells confirming their lymphatic endothelial identity (Fig. 2f, g).

The persistence of the HSC contribution to lymphatic endothelium was determined by evaluating lymphatic vessels for the presence of donor-derived LECs at one month and at greater than one year after transplantation. At these time points, we found a mean of 2.4%±0.8% of lymphatic vessels and 3.2%±1.4% of lymphatic vessels in the portal triad contained GFP+ LECs (Fig. 2i). These donor-derived cells expressed the LEC marker VEGFR-3 (Fig. 2h), demonstrated normal morphology and formed patent vessels whose lumens contained the occasional leukocyte (data not shown). Taken together these findings demonstrate the durable integration of donor HSC-derived LEC into lymphatics.

**Figure 3:**
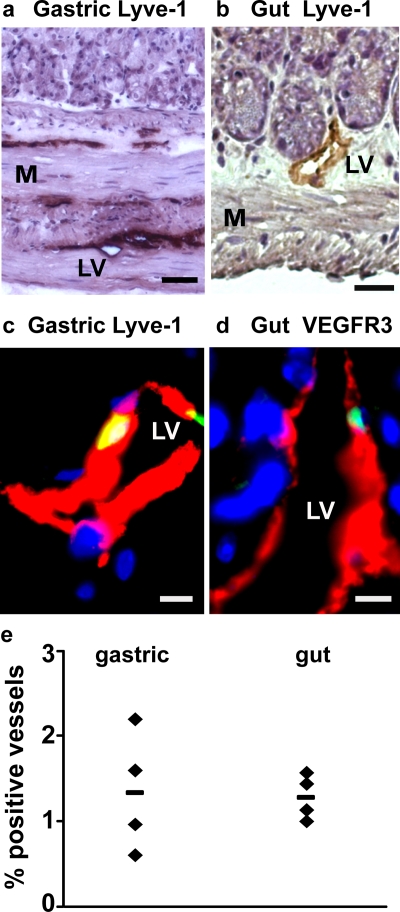
HSC-derived lymphatic endothelial cells are detected in gastrointestinal tissues. (a & b) Bright field images of (a) gastric and (b) gut tissue containing Lyve-1^+^ (brown, DAB-stained) lymphatic vessels. Sections are counterstained with hematoxylin (purple). (c) Merged image of a donor-derived (green), Lyve-1^+^ (red) lymphatic endothelial cell in the stomach. (d) Merged image of donor-derived (green), VEGFR-3+ (red) lymphatic endothelial cell in the gut. DAPI stained nuclei are blue. (e) Frequency of lymphatic vessels in the stomach and gut containing donor-derived endothelial cells after HSC transplantation. Each symbol represents an individual HSC recipient. The horizontal line indicates the average for each group. (LV = Lymphatic Vessel, M = Muscle. Scale bars: a: 50 µm; b: 20 µm; c & d: 5 µm).

To determine the extent to which donor-derived, adult hematopoietic stem cells contribute to lymphatic endothelium in other tissues, the stomach and intestine were investigated in long-term HSC- reconstituted recipients (Fig. 3). A mean of 1.0%±0.7% intestinal lymphatic vessels and 1.4%±0.7% gastric lymphatic vessels were engrafted with GFP^+^ HSC-derived LECs (Fig. 3e). Although the density of lymphatic vessels in the kidney is low, HSC-derived LECs were detected (data not shown). Thus HSCs have the potential to give rise to lymphatic endothelium in a wide range of tissues, including those of both endodermal and mesodermal origin.

### Long-term engraftment of self-renewing HSC with LEC potential

**Figure 4:**
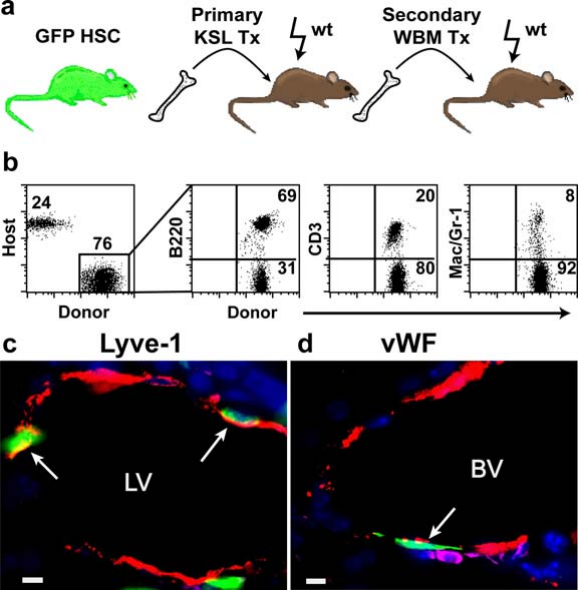
Lymphatic and vascular endothelial potential of self-renewing of HSCs. (a) Secondary transplantation scheme. (b) Flow cytometry analysis of multi-lineage hematopoietic cells in the peripheral blood of secondary recipients. Donor-derived (GFP+) B220 (B-cells), CD3 (T-cells), Mac1/Gr-1(myeloid cells) are shown. (c) Merged image of donor-derived (green), Lyve-1^+^ (red), CD45^−^ (absence of magenta) lymphatic endothelial cells (arrows). (d) Merged image of donor-derived (green), vWF+ (red), CD45- (absence of magenta) vascular endothelial cell (arrow). (DAPI stained nuclei are blue in c,d. LV = lymphatic vessel, BV = blood vessel; scale bars: 5 µm).

In order to directly measure the self-renewal capacity of cells with lymphatic endothelial progenitor activity, serial transplantation studies were performed (Fig. 4a). At 8 months after HSC transplant, whole bone marrow was collected from primary recipients and transplanted into secondary, irradiated recipients. Analysis of the peripheral blood demonstrated high levels of donor HSC-derived, multi-lineage hematopoietic reconstitution (Fig. 4b), confirming that bona-fide HSCs had been transplanted into secondary hosts. Notably, liver lymphatic vessels from these secondary recipients showed clear evidence of donor cell engraftment (Fig. 4c). Donor-derived vascular EC were detected in neighboring liver tissue sections (Fig. 4d), consistent with our previously reported findings [8]. These serial transplantation studies demonstrate that the progeny of donor HSC within the bone marrow have self renewal potential in terms of their capacity to contribute to both blood lineages and lymphatic vascular endothelium.

### Lymphatic endothelium does not arise from a common myeloid progenitor

The ontogenic relationship between venous and lymphatic endothelium raises the possibility that in the adult, a common, circulating bone-marrow derived progenitor might give rise to both lymphatic and vascular endothelial cells. We have recently shown that transplanted common myeloid progenitors (CMPs) and granulocyte/monocyte progenitors (GMPs) contribute to vascular endothelium at frequency similar to that of isolated HSCs [9]. Therefore, we tested whether either of these progenitor populations contributed to lymphatic endothelium in the portal triad area of transplanted mice. Lethally irradiated mice were transplanted with 5–10×10^3^ GFP+ CMPs or GMPs and analyzed 2–3 weeks following transplantation. Although we examined >10,000 lymphatic vessel cross sections from the portal triad region of CMP and GMP recipients, we did not detect any donor-derived lymphatic endothelium. These findings suggest that unlike HSC-derived blood endothelium progenitors, HSC-derived lymphatic progenitors are distinct from the myeloid lineage.

### HSCs contribute to tumor lymphatic endothelium

**Figure 5:**
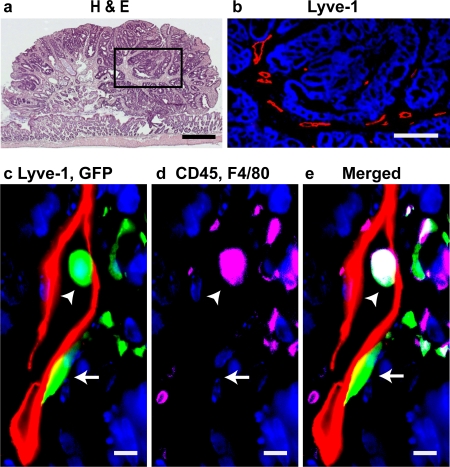
HSC-derived lymphatic endothelial cells incorporate into tumor lymphatics. Following transplantation of HSCs into Min mice, spontaneously developing intestinal tumors were examined for donor-derived (GFP+), Lyve-1^+^, CD45^−^, F4/80^−^ lymphatic endothelial cells. (a) Hematoxylin and eosin (H&E) staining of a tumor in a Min mouse. (b) High magnification of the boxed area in panel a demonstrating Lyve-1+ (red) lymphatic vessels within the tumor. (c) Merged image of a donor-derived (green), Lyve-1+ (red) lymphatic endothelial cell (arrow) and a donor-derived hematopoietic cell (arrow head). (d) The donor-derived hematopoietic cell expresses CD45 and/or F4/80 (magenta), whereas the donor-derived LEC does not. (e) Merged image of c and d (green+ blue+ magenta = white). (DAPI stained nuclei are blue in b–e. Scale bars: a: 500 µm; b: 200 µm, c–e: 5 µm)

Lymphatics are known to be an important contributor to tumor metastasis. To address the question of whether HSCs are capable of contributing to tumor associated lymphatics, we utilized the Multiple Intestinal Neoplasia (Min) mouse model for intestinal tumorigenesis. These mice harbor a mutated *Apc* gene [19] and spontaneously develop multiple intestinal adenomas within the first 8–10 weeks of life [20], [21]. Min mice were irradiated (1200 cGy) and then transplanted with GFP+ HSC cells from Y01 GFP-expressing mice at 5 weeks of age. Six weeks after transplantation, intestinal tumors were harvested and evaluated for endothelial cells that co-expressed CD31 and Lyve-1. Consistent with our findings in normal tissues, blood vessels within these tumors expressed CD31, whereas lymphatic vessels expressed both CD31 (data not shown) and Lyve-1(Fig. 5a,b). In all adenomas examined, we found that donor HSC derived EC also incorporated into the tumor associated lymphatic vessels (Fig. 5c–e). The chimeric lymphatic vessels we observed in the Min mice have a normal, open vessel morphology consistent with the relatively well differentiated state of Min adenomas. Moreover, we detect mononuclear leukocytes within the lymphatic vessel lumen (Fig. 5d). Together, these data suggest that HSC-derived LEC are incorporated in functional lymphatic vessels in these tumors.

### Contribution of donor-derived cells to lymphatic endothelium in the absence of tissue damage

**Figure 6:**
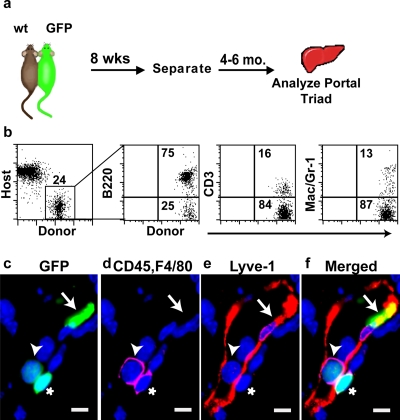
Parabiosis reveals the presence of functional circulating lymphatic progenitors in the absence of acute tissue injury. (a) Non-irradiated wild type mice were surgically joined (parabiosed) with GFP partners. After 12 weeks, mice were separated for between 4–6 months then lymphatic vessels were analyzed. (b) Separated, individual mice displayed stable, donor-derived multi-lineage hematopoietic reconstitution of the peripheral blood. (c–f) Analysis of donor-derived cells in and near a lymphatic vessel. A donor-derived LEC (arrow), a donor-derived hematopoieitc cell within the lymphatic vessel (arrow head), and a donor-derived hematopoietic cell adjacent to the lymphatic vessel (asterisk) are shown. DAPI stained nuclei are blue. Scale bars: 5 µm. (c) Donor-derived, GFP+ cells (green). (d) CD45 and F4/80 distinguishes hematopoietic (magenta, arrowhead and asterisk) from non-hematopoietic (arrow) donor-derived cells. (e) Lyve-1+ (red) reveals the presence of a donor-derived LEC (arrow). (f) Merged image of c–e.

Ionizing radiation is typically employed to induce myeloablation and enhance hematopoietic cell engraftment. However, in addition to ablating the hematopoietic cells, radiation exposure is also known to induce lymphatic endothelial cell apoptosis [22]. This raises the question of whether lymphatic damage might be a prerequisite for the contribution to lymphatic endothelium in our transplant recipients. To address this issue, we employed a parabiosis model and determined whether donor-derived cells can contribute to LECs in the absence of radiation exposure. Specifically, GFP expressing and wild-type mice were surgically joined for 12 weeks, separated, and then analyzed 4–6 months later by flow cytometry and immunohistochemistry (Fig. 6a). As previously reported, wild- type parabiotic mice displayed stable donor-derived (GFP+) multi-lineage hematopoietic reconstitution of the peripheral blood [9], [23]. Circulating, donor-derived B cells, T cells, and myelomonocytic cells were found at the expected frequencies in the parabiotic mice (Fig. 6b). Donor-derived GFP^+^, Lyve-1^+^, CD45^−^, F4/80^−^ lymphatic endothelial cells and GFP+ hematopoietic cells were found in the livers of the parabiotic partners (Fig. 6c–f). In addition, serial transplantation studies of BM harvested from wild-type parabiotic mouse partners confirmed that self-renewing HSCs from the original parabiotic donor were present (data not shown).

## Discussion

In the present study, we show that highly purified, transplanted HSCs can give rise to lymphatic endothelial cells that integrate long-term into lymphatic vessels in many tissues, including the liver, kidney, stomach and gut. Moreover, we demonstrate that HSCs can contribute to LECs associated with intestinal epithelial tumors. Importantly, radiation-induced lymphatic endothelial cell injury is not required for circulating progenitor cells to generate lymphatic endothelium. Our data also demonstrate HSC-derived lymphatic potential is not exclusively found within the myeloid lineage, as transplanted purified myeloid progenitors do not give rise to LECs in vivo. Together, these data suggest that the hematopoietic system can give rise to LECs during steady state conditions, and that this is at least in part mediated by a circulating lymphatic endothelial cell progenitor that is distinct from previously characterized HSC-derived blood vascular endothelial progenitors.

Historically, the formation of new blood vessels (angiogenesis) and lymphatic vessels (lymphangiogenesis) in adults was viewed as driven by the proliferation and remodeling of existing endothelial cells. Recent reports by several groups suggest that bone marrow (BM)-derived hematopoietic cells also have the capacity to contribute to varying degrees to blood vascular endothelium in mouse [8], [9], [10], [11], [24], [25], [26], [27] and humans [12], [13]. Observations such as these have led to the idea that postnatal angiogenesis is driven at least in part by BM-derived endothelial progenitor cells (EPCs), which integrate into and function within neovessels (reviewed by [28]). However, the concept of BM-derived cells with the capacity to produce functional, bona fide ECs is still quite controversial, as some groups have found no evidence to support their existence [29], [30], [31], [32], [33], [34].

In the embryo, LECs are derived from committed venous endothelial cells [7]. However, the origin of adult LECs and their relationship to hematopoietic cells in newly formed lymphatic vessels is controversial. That HSCs are at least one source of lymphatic endothelium is supported by the isolation of hematopoietic cells with LEC potential in vitro [35] and the incorporation of BM- derived cells into lymphatic endothelium [36], [37] as well as into the endothelial cell population at the junction between venous and lymphatic vessels [38]. However, others have found no evidence of BM-derived cell incorporation into lymphatics in their experimental models [39].

It is presently unclear why there is such a disparity in the detection of bone marrow contribution to LEC outcomes. One possibility is that vastly different experimental methods are used to induce lymphangiogenesis, including localized cytokine expression, injection of different tumor cell lines, induction of inflammation and preconditioning with irradiation. Moreover, lymphatics within distinct anatomical locations were analyzed in each of these studies. As there is little known about the molecular heterogeneity of lymphatic endothelium in different organs [2], it is possible that tissue-specific differences in lymphatic induction, growth and repair mechanisms govern the LEC potential of BM-derived cells. Our results support the existence of an HSC-derived lymphatic progenitor that can contribute to a small but significant fraction of lymphatic endothelial cells in the liver, gut and kidney, as well as to lymphatics in spontaneous intestinal adenomas. Moreover, our parabiosis data suggest that neither major injury nor ectopic cytokine induction is necessary for this process to occur.

The methodology used to analyze endothelium is critical for accurately detecting bona fide donor-derived endothelial cells. It has been suggested that perivascular cells, such as macrophages and pericytes, adjacent to vascular endothelial cells may be misidentified as endothelial cells using conventional histological analysis [32], [33]. By logical extension, this argument could be applied to analysis of donor-derived LECs. Importantly, our Z-stack analysis clearly identifies HSC-derived LECs (Fig 2b–e) that do not coexpress macrophage or pericyte markers. Furthermore, unlike vascular endothelium, lymphatic vessels are devoid of pericytes, thereby eliminating these cells as a source of false positive, donor-derived LECs.

In both neoangiogenesis and lymphangiogenesis, a close physical and functional relationship between myeloid cells and endothelium has been observed by many investigators. Tissue macrophages provide paracrine factors that promote endothelial cell proliferation and also serve as scaffolds for neovessels [reviewed by 1], [40], [41], [42]. Myeloid cells also directly contribute to vascular endothelium [9] and lymphatic endothelium [36], [43]. Interestingly, tissue macrophages can transdifferentiate into LECs in vivo, in both inflammatory [36] and wound healing [43] settings. These transient populations of macrophage-derived cells co-express macrophage markers (such as F4/80, used in our studies) and LEC markers. The hypotheses that bone marrow-derived myelomonocytic cells have a strong, non-cell autonomous, cytokine mediated proangiogenic effect and that myelomonocytic progenitor cells can give rise to endothelium are certainly not mutually exclusive. Notably, organ-specific VEGF expression induces angiogenesis by recruiting BM-derived circulating cells, the majority of which are perivascular myeloid cells. In addition, a small fraction of BM-derived cells also incorporate into neovessels. [26]. The extent to which these complementary proangiogenic functions of myelomonocytic cells contribute to normal and pathologic neoangiogenesis remains to be determined.

Given the precedent that myelomonocytic cells can give rise to both vascular ECs and LECs, it is tempting to speculate that all HSC-derived LECs must arise through a myeloid intermediate. However, our analysis of lymphatic vessels within the portal triads of CMP and GMP recipient mice does not support this possibility. We found no evidence of donor cell contribution to lymphatic endothelium in recipients in which we readily detected blood vessels that contained donor CMP- or GMP-derived endothelial cells. Our findings suggest that HSC commitment to the myeloid lineage is not a requisite step in the generation of HSC-derived lymphatic endothelium. However, whether CMPs and GMPs can directly contribute to lymphatic endothelium in other tissues or indirectly contribute to lymphangiogenesis in the setting of tissue inflammation and repair via perivascular myelomonocytic cells remains to be determined.

A close relationship between HSC and lymphatic endothelium has also been demonstrated in a recent study by Massberg and colleagues [44]. They showed that bone marrow derived HSCs with self-renewing activity are present in the lymph of the thoracic duct. Furthermore, their analysis of parabiotic mice revealed that circulating HSCs enter blood circulation and then egress into lymphatics. Based on our findings, we postulate that HSCs present in the lymphatic system may also differentiate into LECs, or alternatively, into a population of functional LEC precursors.

It has been long appreciated that tumor spread into regional lymphatics is an important mechanism of metastasis. Tumor-associated lymphangiogenesis increases lymphatic tumor metastasis in a number of experimental models [1], [2], although fully functional lymphatics within tumors may not be necessary for metastatic spread to regional lymph nodes [45]. Our findings demonstrate that HSC derived LEC are incorporated into tumor associated lymphatic vessels. Future studies will determine whether HSC-derived cells potentiate lymphangiogenesis in tumors and whether targeting HSCs may be a feasible approach for attenuating lymphatic system mediated tumor metastasis.

## Materials and Methods

### Mice

Male donor (8–12 weeks old) C57BL/6-TgN (ACTBEGFP) YO1 mice were harvested for donor cells. Female C57Bl6 recipient mice (8–12 weeks old), and 5-week-old male Min mice [19] were used as recipients. Mice were purchased from the Jackson Laboratory (Bar Harbor, ME) and maintained in a breeding colony in the animal care facility at the Oregon Health & Science University. All procedures were approved by the institutional animal care and use committee of the Oregon Health & Science University.

### Isolation of HSCs and myeloid progenitors

Donor BM was prepared as previously described by Bailey et al [8]. All antibodies were purchased from BD Pharmingen (San Diego, CA) unless otherwise indicated. For KSL isolation, single-cell suspensions of BM were labeled with antibodies to c-kit-allophycocyanin (APC), Ly6 AE (Sca-1) -Biotin with StreptAvidin-PharRed and a phycoerythrin (PE)-conjugated lineage mixture (B220, CD3, CD5, CD4, CD8, Mac-1, Gr-1, Ter119). HSCs were enriched to homogeneity by double sorting c-kit^+^/sca-1^+^/lineage-negative (lin^−^) cells using a fluorescence-activated cell sorter (FACS) Vantage. Myeloid progenitors were isolated as described previously [9]. Bone marrow cells were labeled with a PE-conjugated lineage mixture (B220, CD3, CD4, CD8, Gr-1,CD19, IgM [e-biosciences, San Diego, CA], and Ter119) and the antibodies IL-7Rα-PE (e-biosciences), Ly6AE-PE, c-kit-APC-Cy7(e-biosciences), CD34-FITC, and FcγRII/III-APC (e-biosciences). Purified progenitors were obtained by double-sorting of IL-7Rα^−^ Lin^−^Sca-1^−^c kit^+^CD34^+^FcγRII/III^lo^ (CMP) and IL-7Rα^−^ Lin^−^ Sca-1^−^ c- kit^+^ CD34^+^ FcγRII/III^hi^ (GMP) populations. Dead cells were excluded by a combination of scatter gates and propidium iodide staining.

### Transplantation studies

Recipient mice were treated with 1200 cGy of lethal irradiation in 2 equal doses of 600 cGy delivered 3 hours apart. The indicated number of donor cells was injected intravenously into the retro-orbital plexus. Recipients received antibiotic water for one month after transplantation (neomycin sulfate at 1.1 g/L and polymyxin B sulfate at 167 mg/L).

### Parabiosis

Parabiotic pairs of 6- to 12-week-old age- and weight-matched female WT and Y01 GFP+ mice were made as previously described [9]. Each parabiotic recipient was given recombinant human granulocyte colony-stimulating factor (250 mg/kg s.c.) for 4 days at 2–3 weeks after surgery. Peripheral blood was collected and analyzed by flow cytometry to evaluate donor chimerism through cross-circulation. Mice were separated 12 weeks after surgery, and peripheral blood and liver were analyzed 4–6 months later.

### Hematopoietic Engraftment

Peripheral blood leukocytes were obtained after erythrocyte depletion by sedimentation in 3% dextran (Amersham Pharmacia, Uppsala, Sweden) and hypotonic lysis. Multi-lineage hematopoietic engraftment was analyzed with the antibodies CD45.2-FITC (donor), CD45.1-PE (host), and the lineage markers Mac1-APC, Gr1-APC, B220- APC, and CD3-APC as previously described [46].

### Immunohistochemistry

Recipient mice were sacrificed between 1 and 12 month after transplantation. Liver, kidney, stomach and gut were fixed in 4% paraformaldehyde for 4 hours, washed, dehydrated in 30% sucrose, then cryopreserved in optimal cutting temperature (OCT). In some instances, tissues were fixed formaldehyde with zinc buffer (Z-fix; Anatech, Battle Creek, MI), embedded in paraffin and sectioned. Five micrometer tissue sections were incubated overnight at 4°C with rabbit anti-LYVE-1 (1∶800; Upstate Biotechnology, Lake Placid, NY), rat anti-CD31 (1∶50; BD Pharmingen) or rat anti-VEGFR3 (1∶25; BD Pharmingen), followed by a Cy3 conjugated secondary antibody (Chemicon, Temecula, CA). To differentiate donor-derived hematopoietic cells, we double stained the sections with rat anti-CD45 (1∶50; BD Pharmingen) and rat anti-F4/80 (1∶100; Serotec, Raleigh, NC), followed by Alexafluor 647 chicken anti-rat (Invitrogen-Molecular Probes, Eugene, OR). The nuclei were counterstained with DAPI (4,6 diamidino-2-phenylindole). Tyramide signal amplification (NEN Life Science, Boston, MA) was employed to enhance VEGFR-3 staining in paraffin sections, using the Cy3 tyramide reagent. Rat IgG2a κ or rat IgG2b κ was used as a negative control for CD31, CD45 and F4/80 antibody staining.

### Image Analysis

Sections were examined and photographed with a Zeiss Axiophot 200 microscope by using a true color AxioCam camera and standard epifluorescence filters for fluorescein isothiocyanate (FITC), Cy3, Cy5 and DAPI (Carl Zeiss, Jena, Germany). Images were digitally combined using Axio Vision software (Carl Zeiss). Z-stack images were obtained with a BioRad 1024 ES confocal laser scanning microscope (BioRad, Hercules, CA) and analyzed using Delta Vision deconvolution software (BioRad). A minimum of 100 liver lumen-containing lymphatic vessels (based on LYVE-1 staining) were counted per recipient mouse.

## References

[1] Ji RC (2007). Lymphatic endothelial cells, inflammatory lymphangiogenesis, and prospective players.. Curr Med Chem.

[2] Karpanen T, Alitalo K (2008). Molecular Biology and Pathology of Lymphangiogenesis.. Annu Rev Pathol.

[3] Liersch R, Detmar M (2007). Lymphangiogenesis in development and disease.. Thromb Haemost.

[4] Achen MG, Stacker SA (2006). Tumor lymphangiogenesis and metastatic spread-new players begin to emerge.. Int J Cancer.

[5] Alitalo K, Tammela T, Petrova TV (2005). Lymphangiogenesis in development and human disease.. Nature.

[6] Sundar SS, Ganesan TS (2007). Role of lymphangiogenesis in cancer.. J Clin Oncol.

[7] Srinivasan RS, Dillard ME, Lagutin OV, Lin FJ, Tsai S (2007). Lineage tracing demonstrates the venous origin of the mammalian lymphatic vasculature.. Genes Dev.

[8] Bailey AS, Jiang S, Afentoulis M, Baumann CI, Schroeder DA (2004). Transplanted adult hematopoietic stems cells differentiate into functional endothelial cells.. Blood.

[9] Bailey AS, Willenbring H, Jiang S, Anderson DA, Schroeder DA (2006). Myeloid lineage progenitors give rise to vascular endothelium.. Proc Natl Acad Sci U S A.

[10] Grant MB, May WS, Caballero S, Brown GA, Guthrie SM (2002). Adult hematopoietic stem cells provide functional hemangioblast activity during retinal neovascularization.. Nat Med.

[11] Larrivee B, Niessen K, Pollet I, Corbel SY, Long M (2005). Minimal contribution of marrow-derived endothelial precursors to tumor vasculature.. J Immunol.

[12] Jiang S, Walker L, Afentoulis M, Anderson DA, Jauron-Mills L (2004). Transplanted human bone marrow contributes to vascular endothelium.. Proc Natl Acad Sci U S A.

[13] Peters BA, Diaz LA, Polyak K, Meszler L, Romans K (2005). Contribution of bone marrow-derived endothelial cells to human tumor vasculature.. Nat Med.

[14] Banerji S, Ni J, Wang SX, Clasper S, Su J (1999). LYVE-1, a new homologue of the CD44 glycoprotein, is a lymph-specific receptor for hyaluronan.. J Cell Biol.

[15] Kaipainen A, Korhonen J, Mustonen T, van Hinsbergh VW, Fang GH (1995). Expression of the fms-like tyrosine kinase 4 gene becomes restricted to lymphatic endothelium during development.. Proc Natl Acad Sci U S A.

[16] Dudas J, Papoutsi M, Hecht M, Elmaouhoub A, Saile B (2004). The homeobox transcription factor Prox1 is highly conserved in embryonic hepatoblasts and in adult and transformed hepatocytes, but is absent from bile duct epithelium.. Anat Embryol (Berl).

[17] Schledzewski K, Falkowski M, Moldenhauer G, Metharom P, Kzhyshkowska J (2006). Lymphatic endothelium-specific hyaluronan receptor LYVE-1 is expressed by stabilin-1+, F4/80+, CD11b+ macrophages in malignant tumours and wound healing tissue in vivo and in bone marrow cultures in vitro: implications for the assessment of lymphangiogenesis.. J Pathol.

[18] Lamagna C, Bergers G (2006). The bone marrow constitutes a reservoir of pericyte progenitors.. J Leukoc Biol.

[19] Moser AR, Pitot HC, Dove WF (1990). A dominant mutation that predisposes to multiple intestinal neoplasia in the mouse.. Science.

[20] Moser AR, Dove WF, Roth KA, Gordon JI (1992). The Min (multiple intestinal neoplasia) mutation: its effect on gut epithelial cell differentiation and interaction with a modifier system.. J Cell Biol.

[21] Roy HK, Kim YL, Wali RK, Liu Y, Koetsier J (2005). Spectral markers in preneoplastic intestinal mucosa: an accurate predictor of tumor risk in the MIN mouse.. Cancer Epidemiol Biomarkers Prev.

[22] Abtahian F, Guerriero A, Sebzda E, Lu MM, Zhou R (2003). Regulation of blood and lymphatic vascular separation by signaling proteins SLP-76 and Syk.. Science.

[23] Abkowitz JL, Robinson AE, Kale S, Long MW, Chen J (2003). Mobilization of hematopoietic stem cells during homeostasis and after cytokine exposure.. Blood.

[24] Asahara T, Masuda H, Takahashi T, Kalka C, Pastore C (1999). Bone marrow origin of endothelial progenitor cells responsible for postnatal vasculogenesis in physiological and pathological neovascularization.. Circ Res.

[25] Crosby JR, Kaminski WE, Schatteman G, Martin PJ, Raines EW (2000). Endothelial cells of hematopoietic origin make a significant contribution to adult blood vessel formation.. Circ Res.

[26] Grunewald M, Avraham I, Dor Y, Bachar-Lustig E, Itin A (2006). VEGF-induced adult neovascularization: recruitment, retention, and role of accessory cells.. Cell.

[27] Lyden D, Hattori K, Dias S, Costa C, Blaikie P (2001). Impaired recruitment of bone-marrow-derived endothelial and hematopoietic precursor cells blocks tumor angiogenesis and growth.. Nat Med.

[28] Ribatti D (2007). The discovery of endothelial progenitor cells. An historical review.. Leuk Res.

[29] De Palma M, Venneri MA, Galli R, Sergi Sergi L, Politi LS (2005). Tie2 identifies a hematopoietic lineage of proangiogenic monocytes required for tumor vessel formation and a mesenchymal population of pericyte progenitors.. Cancer Cell.

[30] De Palma M, Venneri MA, Roca C, Naldini L (2003). Targeting exogenous genes to tumor angiogenesis by transplantation of genetically modified hematopoietic stem cells.. Nat Med.

[31] Gothert JR, Gustin SE, van Eekelen JA, Schmidt U, Hall MA (2004). Genetically tagging endothelial cells in vivo: bone marrow-derived cells do not contribute to tumor endothelium.. Blood.

[32] Purhonen S, Palm J, Rossi D, Kaskenpaa N, Rajantie I (2008). Bone marrow-derived circulating endothelial precursors do not contribute to vascular endothelium and are not needed for tumor growth.. Proc Natl Acad Sci U S A.

[33] Rajantie I, Ilmonen M, Alminaite A, Ozerdem U, Alitalo K (2004). Adult bone marrow-derived cells recruited during angiogenesis comprise precursors for periendothelial vascular mural cells.. Blood.

[34] Ziegelhoeffer T, Fernandez B, Kostin S, Heil M, Voswinckel R (2004). Bone marrow-derived cells do not incorporate into the adult growing vasculature.. Circ Res.

[35] Salven P, Mustjoki S, Alitalo R, Alitalo K, Rafii S (2003). VEGFR-3 and CD133 identify a population of CD34+ lymphatic/vascular endothelial precursor cells.. Blood.

[36] Maruyama K, Ii M, Cursiefen C, Jackson DG, Keino H (2005). Inflammation-induced lymphangiogenesis in the cornea arises from CD11b-positive macrophages.. J Clin Invest.

[37] Religa P, Cao R, Bjorndahl M, Zhou Z, Zhu Z (2005). Presence of bone marrow-derived circulating progenitor endothelial cells in the newly formed lymphatic vessels.. Blood.

[38] Sebzda E, Hibbard C, Sweeney S, Abtahian F, Bezman N (2006). Syk and Slp-76 mutant mice reveal a cell-autonomous hematopoietic cell contribution to vascular development.. Dev Cell.

[39] He Y, Rajantie I, Ilmonen M, Makinen T, Karkkainen MJ (2004). Preexisting lymphatic endothelium but not endothelial progenitor cells are essential for tumor lymphangiogenesis and lymphatic metastasis.. Cancer Res.

[40] Kerjaschki D (2005). The crucial role of macrophages in lymphangiogenesis.. J Clin Invest.

[41] Murdoch C, Muthana M, Coffelt SB, Lewis CE (2008). The role of myeloid cells in the promotion of tumour angiogenesis.. Nat Rev Cancer.

[42] Shojaei F, Zhong C, Wu X, Yu L, Ferrara N (2008). Role of myeloid cells in tumor angiogenesis and growth.. Trends Cell Biol.

[43] Maruyama K, Asai J, Ii M, Thorne T, Losordo DW (2007). Decreased macrophage number and activation lead to reduced lymphatic vessel formation and contribute to impaired diabetic wound healing.. Am J Pathol.

[44] Massberg S, Schaerli P, Knezevic-Maramica I, Kollnberger M, Tubo N (2007). Immunosurveillance by hematopoietic progenitor cells trafficking through blood, lymph, and peripheral tissues.. Cell.

[45] Padera TP, Kadambi A, di Tomaso E, Carreira CM, Brown EB (2002). Lymphatic metastasis in the absence of functional intratumor lymphatics.. Science.

[46] Montfort MJ, Olivares CR, Mulcahy JM, Fleming WH (2002). Adult blood vessels restore host hematopoiesis following lethal irradiation.. Exp Hematol.

